# Genomic Characterization of Clinical Group B *Streptococcus* Isolates from Zhejiang, China: Serotypes, Clonal Complexes, and Predicted Antimicrobial Resistance and Virulence Gene Profiles

**DOI:** 10.3390/microorganisms14102284

**Published:** 2026-10-08

**Authors:** Zhangnv Yang, Zi Jin, Lingbo Wang, Guoxiang Shi, Wenwu Yao, Xiaowei Gong, Beibei Wu, Weilin Hu

**Affiliations:** 1 Key Lab of Vaccine, Prevention and Control of Infectious Disease of Zhejiang Province, Zhejiang Provincial Center for Disease Control and Prevention, Hangzhou 310015, China; znyang@cdc.zj.cn (Z.Y.); zjin@cdc.zj.cn (Z.J.); lbwang@cdc.zj.cn (L.W.);; 2 Department of Microbiology, Zhejiang University School of Medicine, Hangzhou 310058, China

**Keywords:** GBS, WGS, resistance genes, virulence genes

## Abstract

This study aimed to characterize the population structure, predicted antimicrobial resistance gene profiles, and virulence gene repertoire of Group B *Streptococcus* (GBS) in Zhejiang Province, China, using whole-genome sequencing (WGS). Forty GBS isolates from clinical specimens underwent WGS, multilocus sequence typing (MLST), cgMLST-based phylogenetic analysis, and systematic in silico annotation of predicted antimicrobial resistance and virulence genes. Seven capsular serotypes were identified, with serotype V being predominant. Fifteen sequence types (STs) were detected, including two novel STs (ST2662 and ST2663). Thirteen STs were grouped into eight clonal complexes (CCs), while two STs remained unassigned to any CC. CC19 (11/40, 27.5%) and CC327 (10/40, 25.0%) were the predominant CCs, with CC327 comprising the distinctive ST529-CC327-V clone. Eighteen predicted antimicrobial resistance genes were identified, including genes predicted to confer resistance to macrolide (*ermB* 60.0%, *mefA* 37.5%), tetracycline (*tetM* 47.5%, *tetO* 42.5%), aminoglycoside (*aph*(*3′*)*-IIIa* 25.0%), and lincosamide (*lsaE* 30.0%, *lnuB* 30.0%). All isolates carried multiple predicted virulence genes, with universal carriage of adhesion genes *fbsA* and *fbsB*, and the invasion genes *cylE*, *hylB*, and *cfb*. These findings reveal a genetically diverse GBS population with distinctive clonal architecture and high carriage of predicted antimicrobial resistance genes in Zhejiang Province, underscoring the need for continued genomic surveillance to inform region-specific antibiotic stewardship and vaccine development.

## 1. Introduction

Group B *Streptococcus* (GBS), also known as *S. agalactiae*, is an important perinatal pathogen and a leading cause of neonatal sepsis, pneumonia, and meningitis in neonates, as well as endometritis, chorioamnionitis, puerperal sepsis, and urinary tract infections in pregnant and postpartum women [1]. In recent years, the detection rate of adult GBS infections has increased steadily, posing a substantial health threat to immunocompromised individuals and elderly populations worldwide [2,3]. These infections manifest as a broad clinical spectrum, including urogenital, bloodstream, and skin and soft-tissue infections, and are complicated by substantial genetic heterogeneity among strains, challenging clinical prevention and antimicrobial management [4,5].

Previous studies have predominantly focused on GBS isolates from vaginal specimens of pregnant women, with major attention paid to molecular characteristics related to vertical mother-to-child transmission [6,7]. However, genomic data from non-pregnant populations, male patients, and non-genital clinical specimens (such as urine and blood) remain scarce. This gap in understanding has limited region-specific insights into clinical GBS infection characteristics in diverse patient groups.

Given the above research gaps, we performed a retrospective genomic analysis of 40 clinical GBS isolates from multiple hospitals in Zhejiang Province, encompassing non-pregnant adults, male individuals, and elderly patients, as well as diverse clinical specimen types including urine and blood. Whole-genome sequencing was used to describe the genomic profiles of these isolates. This study sought to describe the distribution of sequence types (STs), clonal complexes (CCs), and serotypes, and to profile the carriage of predicted virulence factors and antimicrobial resistance genes. The findings may contribute to a better understanding of regional GBS population structure and predicted antimicrobial resistance and virulence gene patterns, informing clinical antibiotic stewardship and highlighting priorities for future surveillance in this region.

## 2. Materials and Methods

### 2.1. Bacterial Isolates and DNA Preparation

A total of 40 non-duplicate clinical isolates suspected to be GBS were collected from sentinel hospitals across Zhejiang Province between March 2024 and November 2025. The data were kept in the Zhejiang Provincial Intelligent Preservation Platform for Pathogenic Microorganism Specimen Bank and Culture (Virus) Collection System. At the time of original isolation, strains were streaked onto blood agar plates without any supplements or selective antibiotics (CHROMagar, Shanghai, China), incubated at 37 °C for 18 h under 5% CO_2_, and purified by subculture. Species identification was confirmed by matrix-assisted laser desorption/ionization time-of-flight mass spectrometry (MALDI-TOF MS; Bruker Corporation, Karlsruhe, Germany). Genomic DNA was subsequently extracted, archived at −80 °C, and used retrospectively in the present study for whole-genome sequencing.

### 2.2. Genome Sequencing and Assembly

Genomic DNA of all *Streptococcus agalactiae* isolates was extracted using the QIAamp DNA Mini Kit (Qiagen, Hilden, Germany) following the manufacturer’s instructions. DNA concentration and purity were assessed by spectrophotometry (NanoDrop, Thermo Fisher Scientific, Waltham, MA, USA); samples with an A260/A280 ratio of 1.8 to 2.0 were used for whole-genome sequencing.

Whole-genome sequencing was performed by using MGIEasy FS DNA Library Prep Set V2.0 on the MGISEQ-2000RS platform (MGI Tech Co., Ltd., Shenzhen, China), generating 150-bp paired-end reads with an average coverage depth exceeding 100×. For data processing, the reads quality was analyzed with FastQC v0.12.1 (https://github.com/s-andrews/FastQC, accessed on 15 May 2026) and processed using Trimmomatic v0.39 (https://github.com/timflutre/trimmomatic, accessed on 20 May 2026) to eliminate low-quality sequence ends and sequencing adapter contamination. Reads after quality control were used for species identification using Kraken2 v2.1.6 (https://github.com/DerrickWood/kraken2, accessed on 9 June 2026) and for de novo assembly using Unicycler v0.4.8 (https://github.com/rrwick/Unicycler, accessed on 14 June 2026). The quality of the de novo assemblies was assessed using QUAST v5.2.0 (https://github.com/ablab/quast, accessed on 21 June 2026), and high-quality genomes (completeness > 95%, contamination < 5%) were retained for downstream analyses. Genome assembly quality statistics were provided as Supplementary Table S1.

### 2.3. In Silico Analysis

Reference sequences representing the ten serotypes (Ia-IX) against the capsular polysaccharide were obtained according to the previous study [8], then alignment-based calling of serotypes was done using BLASTn between the reference sequences and sequences of 40 isolated strains. A minimum nucleotide identity of 95% across the 90% of the reference serotype length was required for making a serotype call, and assignments below these thresholds were reported as non-typeable (NT).

The multilocus sequence typing (MLST) of the genomes was screened against the seven housekeeping gene loci of *S. agalactiae*, including *adhP*, *atr*, *glcK*, *glnA*, *pheS*, *sdhA*, *tkt* by querying the assembled genomes against the PubMLST *S. agalactiae* database (https://pubmlst.org/sagalactiae, accessed on 5 September 2026) to assign sequence types (STs), and clonal complexes (CCs) were determined according to the database definitions, in which STs are grouped into a CC when they share at least 6 of the 7 MLST alleles with at least one other member of the group.

Predicted antimicrobial resistance (AMR) genes were annotated by aligning genomic sequences against the Comprehensive Antibiotic Resistance Database (CARD; https://card.mcmaster.ca, accessed on 9 June 2026) using Resistance Gene Identifier (RGI) software (version 5.1.0). Matches were processed as ≥95% identity and ≥80% coverage. Virulence factors were predicted by BLAST v.2.17.0 analysis of genomic sequences against the Virulence Factor Database (VFDB, accessed on 9 June 2026). Predicted virulence factors were retained if they exhibited ≥75% sequence identity and ≥85% coverage with an E-value < 1 × 10^−5^.

For phylogenetic analysis, a total of 40 isolates from the present study and 70 reference sequences representing different lineages (retrieved from the NCBI GenBank database on 15 April 2026, country of origin, serotype, ST and CC are listed in Supplementary Table S2) were included. During the selection process, strains with complete information were preferred (with high-quality drafts accepted where no complete genome was available for a lineage). Subsequently, genomes were selected to span the major human-associated clonal complexes to include isolates from different countries (with Asian/Chinese isolates preferentially retained), and to represent distinct STs. To avoid over-representation of heavily sampled lineages, no more than five genomes were included per sequence type, with genomes otherwise randomly selected within each clonal complex from among those meeting the above criteria. Comprehensive genome analysis was conducted using cgMLST (core genome multilocus sequence typing) Target Definer module of Ridom© SeqSphere+ software v5.0.2 (Ridom GmbH, Münster, Germany) containing 1770 targets according to the software instruction. Allele calling was performed for all 110 genomes. For each pair of genomes, the allele distance was calculated as the number of the 1770 loci at which the two genomes carried different alleles (allelic mismatches),loci lacking an allele call in both genomes of a pair were handled by pairwise deletion, the single missing locus was ignored in the corresponding pairwise distance calculation.

The remaining strains were used to construct a phylogenetic tree using the neighbor-joining (NJ) algorithm in SeqSphere+ software. As NJ is a distance-based method operating on the allele-distance matrix rather than on a nucleotide sequence alignment, no nucleotide substitution model was applied; the distance metric described above (number of differing cgMLST alleles) is the corresponding parameter. Branch support was not calculated, and the tree was treated as unrooted.

### 2.4. Statistical Analysis

Given the modest sample size (*n* = 40) and uneven distribution across subgroups, no formal statistical inference was performed. All subgroup observations, including comparisons between ST19 and ST529, are presented as descriptive frequencies and percentages only. No *p*-values were calculated.

### 2.5. Heatmap Construction and Hierarchical Clustering

Predicted antimicrobial resistance and virulence gene profiles were converted into a binary presence/absence matrix, with gene presence coded as 1 and absence as 0 (Table S3). Missing values were treated as absence, and no additional normalization or scaling was applied. Hierarchical clustering was performed using Euclidean distance and complete-linkage agglomeration. Predicted virulence and antimicrobial resistance genes were separated into two functional groups and clustered independently within each group. Isolates were grouped according to ST, CC, or serotype, depending on the corresponding heatmap, and hierarchical clustering was performed within each group. Heatmaps were generated in R v4.4.1 using the ComplexHeatmap package, with color mapping implemented using the circlize package.

## 3. Results

### 3.1. Clinical Distribution of GBS Isolates

This retrospective study included 40 non-duplicate clinical GBS isolates originally recovered from multiple sentinel hospitals across Zhejiang Province between March 2024 and November 2025. As summarized in Table 1, the isolates were obtained from 28 (70.0%) female and 12 (30.0%) male patients. By age group, 13 (32.5%) isolates were from patients aged 18–39 years, 12 (30.0%) from 40–59 years, 13 (32.5%) from ≥60 years, and 2 (5.0%) from 0–17 years. Urine was the predominant specimen type (17/40, 42.5%), followed by secretion (14/40, 35.0%), blood (5/40, 12.5%), and tissue fluid (1/40, 2.5%). The specimen source was not recorded for 3 isolates (7.5%) due to incomplete clinical documentation. The clinical metadata was provided as Supplementary Table S4.

### 3.2. Population Structure: Serotypes, STs, and CCs

**Figure 1:**
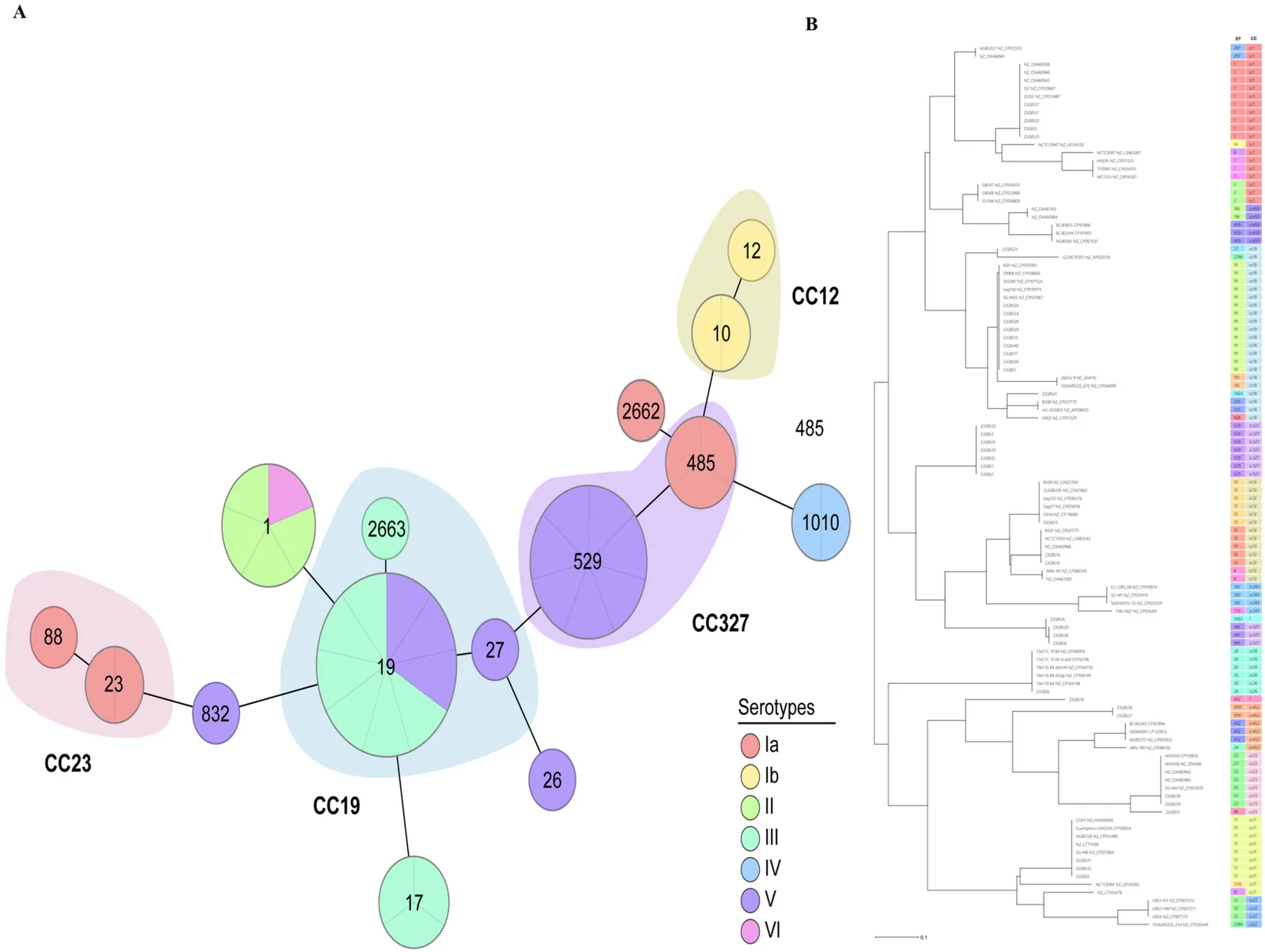
Population structure and phylogenetic relationships of clinical GBS isolates. (**A**) Minimum spanning tree of 40 ST-typed isolates. The numbers in the circle stand for the ST (Sequence type), the colors in the circle stand for the serotype, the shadow color stands for the CC (Clonal complex). (**B**) Neighbor-joining phylogenetic tree of 40 clinical isolates and 70 reference strains based on cgMLST allelic profiles containing 1770 targets. ST, Sequence type; CC, Clonal complex.

The 40 isolates were assigned to seven capsular serotypes. Serotype V was the most prevalent (13/40, 32.5%), followed by III (10/40, 25.0%), Ia (7/40, 17.5%), II (4/40, 10.0%), Ib (3/40, 7.5%), IV (2/40, 5.0%), and VI (1/40, 2.5%) (Table 2). Multilocus sequence typing identified 13 STs among 38 isolates. For the remaining two isolates that were initially untypeable, we submitted them to PubMLST *S. agalactiae* database, where they were assigned as two novel sequence types, ST2662 and ST2663. Thus, all 40 isolates were assigned to 15 STs in total (Table 3). ST19 was the most prevalent (9/40, 22.5%), followed by ST529 (7/40, 17.5%), ST1 (5/40, 12.5%), and ST17 and ST485 (3/40 each, 7.5%). Minimum spanning tree analysis grouped the 40 isolates into eight CCs, with two STs remaining unassigned to any CC (Figure 1A, Table 3). CC19 was the predominant complex, comprising 11 isolates (27.5%), followed by CC327 with 10 isolates (25.0%). Notably, all seven ST529 isolates were assigned exclusively to CC327 and uniformly carried serotype V, the most prevalent serotype–ST combination in this study. To contextualize the genetic diversity of the clinical isolates, a neighbor-joining phylogenetic tree was constructed based on cgMLST allelic profiles of the 40 study isolates together with 70 reference strains retrieved from the NCBI GenBank database (Figure 1B). The 110 strains were divided into two major lineages, with the clinical isolates distributed across both branches.

### 3.3. Predicted Antimicrobial Resistance Genes

**Figure 2:**
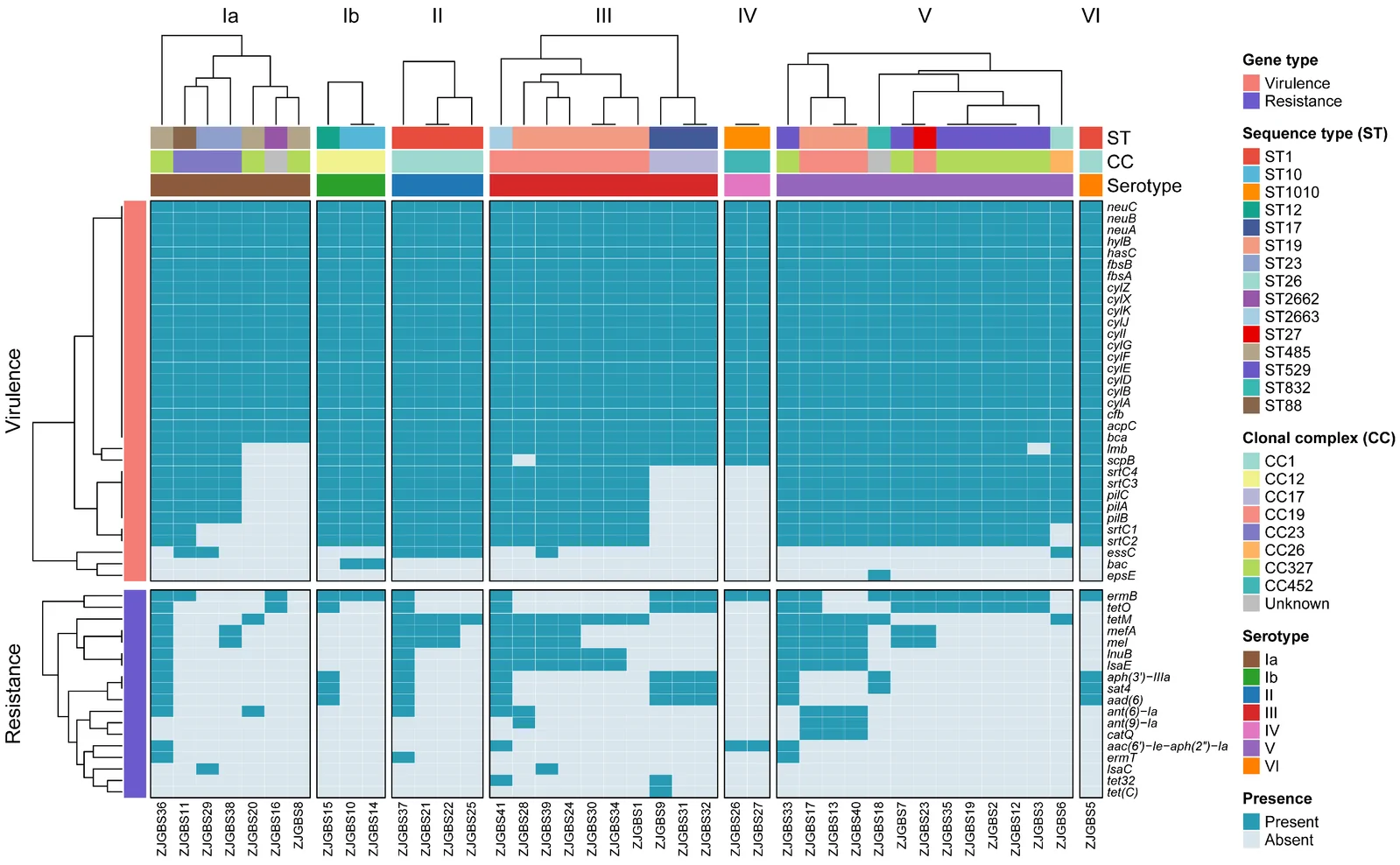
Distribution and prevalence of predicted antimicrobial resistance and virulence genes among 40 GBS isolates.

Predicted antimicrobial resistance genes were screened in all 40 GBS isolates. A total of 18 predicted resistance determinants were identified, including those conferring resistance to aminoglycosides [*aac*(*6′*)*-Ie-aph*(*2″*)*-Ia*, *aad*(*6*), *ant*(*6*)*-Ia*, *ant*(*9*)*-Ia*, and *aph*(*3′*)*-IIIa*], macrolides (*ermB*, *ermT*, *mefA*, and *mel*), tetracyclines (*tetO*, *tetM*, *tet32*, and *tet*(*C*)), phenicols (*catQ*) and lincosamides (*lsaC*, *lnuB*, and *lsaE*), and streptothricin (*sat4*) (Figure 2). Among predicted macrolide resistance genes, *ermB* was the most prevalent (24/40, 60.0%), followed by *mefA* and *mel* (15/40 each, 37.5%). Predicted tetracycline resistance genes *tetM* and *tetO* were detected in 19 (47.5%) and 17 (42.5%) isolates, respectively, whereas *tet32* and *tet*(*C*) were less common. The predicted aminoglycoside resistance genes *aph*(*3′*)*-IIIa*, *aad*(*6*), and *ant*(*6*)*-Ia* were identified in 10 (25.0%), 9 (22.5%), and 8 (20.0%) isolates, respectively; the predicted lincosamide resistance genes *lsaE* and *lnuB* were each found in 12 isolates (30.0%); and *sat4*, which confers resistance to streptothricin, was present in 8 (20.0%).

Hierarchical clustering based on predicted resistance gene profiles revealed distinct genotypic patterns that varied by serotype, ST, and CC. ST19 and ST529, the two most common sequence types, showed distinct resistance gene carriage patterns: ST19 isolates frequently carried *tetM* (100.0%, 9/9), *lnuB* (88.9%, 8/9), and *lsaE* (88.9%, 8/9) but rarely carried *ermB* or *tetO* (11.1%, 1/9 each), whereas ST529 isolates frequently carried *ermB* and *tetO* (100.0%, 7/7) but rarely carried *tetM*, *lnuB*, or *lsaE* (14.3%, 1/7 each). These differences are presented descriptively because of the small subgroup sizes. CC17 exhibited co-carriage of *ermB*, *tetO*, and the aminoglycoside resistance gene cluster *aph*(*3′*)*-IIIa*/*sat4*/*aad*(*6*) at 100.0% (3/3), a descriptive observation in this small lineage. Serotype V showed the highest rates of *ermB* (76.9%, 10/13) and *tetO* (69.2%, 9/13) and also carried *ant*(*6*)*-Ia*, *ant*(*9*)*-Ia*, and *catQ*, whereas serotype III carried *tetM* (70.0%, 7/10), *lnuB* (60.0%, 6/10), and *lsaE* (60.0%, 6/10), and serotype II harbored *tetM* at 100.0% (4/4), a descriptive observation given the small sample size.

### 3.4. Distribution of Predicted Virulence Genes

All virulence gene data reported in this section are based on in silico presence/absence annotation and do not include locus-level validation or mutation screening within genes; they therefore represent predicted genomic potential rather than confirmed functional activity. Based on whole-genome sequencing, systematic annotation and distribution analysis of predicted virulence genes were performed across 40 GBS isolates. As shown in Figure 2, the tested strains harbored multiple classes of genes predicted to encode virulence factors associated with adhesion, invasion, and immune evasion, with a high overall carriage rate. Notably, the genes *fbsA* and *fbsB*, predicted to encode fibrinogen-binding proteins, were each present in all 40 isolates (100.0%). The genes *scpB* and *lmb*, predicted to encode C5a peptidase and laminin-binding protein, respectively, were each detected at carriage rates of 90.0% (36/40). The genes *pilA*, *pilB*, and *pilC*, predicted to encode pilus structural proteins, were each detected at carriage rates of 80.0% (32/40). The genes *cylE*, *hylB*, *bca*, and *cfb*, predicted to encode β-hemolysin/cytolysin, hyaluronate lyase, alpha-C protein, and CAMP factor, respectively, were present in all isolates. Additionally, the sialic acid biosynthesis genes *neuA*, *neuB*, and *neuC*, which are predicted to contribute to capsular sialylation, were detected in 100% of the isolates. Hierarchical clustering of virulence gene profiles partitioned the isolates into distinct genotypic patterns consistent with their serotype, ST, and CC designations. The *bac* gene, predicted to encode β-C protein (Bac), was present only in ST10-CC12-Ib isolates (2/2, 100.0%). ST1-CC1-II strains universally harbored the *essC* gene (100%, 4/4). No hits were obtained for the generic markers *srtC1/C2/C3/C4*, *pilA/B/C*, and *essC* in ST17-CC17 (*n* = 3) and ST1010-CC452 (*n* = 2) isolates.

## 4. Discussion

This retrospective genomic study of 40 clinical GBS isolates from Zhejiang Province revealed a population dominated by capsular serotype V, with CC19 and CC327 as the predominant clonal complexes. The isolates carried a substantial burden of predicted antimicrobial resistance determinants, particularly genes predicted to confer macrolide and tetracycline resistance, and showed near-universal carriage of virulence factors mediating adhesion, invasion, and immune evasion. These findings contribute to a growing body of evidence that clinical GBS populations in eastern China are genetically diverse, genotypically enriched for predicted antimicrobial resistance, and harbor diverse virulence determinants.

The demographic and clinical profile of the isolates in this study, encompassing non-pregnant adults, male patients, and elderly individuals, with urine and non-genital secretions as predominant sources, reflects an expanding host and clinical spectrum beyond the traditional obstetric and neonatal contexts. Invasive GBS disease among non-pregnant adults, particularly those aged ≥65 years or with immunocompromising conditions, has risen substantially in North America and Europe over the past two decades [9,10,11]. The high proportion of elderly patients (32.5%) and the recovery of isolates from blood and tissue fluid in the present cohort are consistent with this global epidemiological shift, underscoring the need to broaden genomic surveillance beyond perinatal populations.

GBS is classified into 10 distinct capsular polysaccharide-based serotypes, and serotypes Ia, Ib, II, III, and V represent the predominant circulating strains, collectively accounting for more than 90% of clinical isolates [12]. In the present study, capsular serotype V was the most prevalent type (32.5%), followed by serotypes III (25.0%) and Ia (17.5%). Serotype V has been identified as one of the most frequent serotypes in invasive adult disease, accounting for a prominent proportion of urogenital tract infections and skin and soft-tissue infections, and is often associated with CC1 and CC19 lineages [13]. In contrast, serotype III demonstrates the highest pathogenicity and is the leading causative agent of neonatal meningitis [14]. The co-dominance of III and V in the present cohort suggests that the circulating GBS population in Zhejiang retains virulent lineages capable of causing both neonatal and adult invasive infections.

MLST defines sequence types as the standardized molecular classification scheme for GBS. To date, over 2600 distinct STs have been identified worldwide (https://pubmlst.org/sagalactiae, accessed on 5 September 2026), which are closely linked to capsular serotypes, clonal complexes, virulence determinants, and antimicrobial resistance profiles [15,16], establishing MLST as a cornerstone for epidemiological surveillance and infection source tracking. ST distribution exhibits marked geographic heterogeneity. In Europe, ST19, ST23, and ST1 represent the predominant lineages [17], whereas ST283, ST1, and ST17 are the most frequently encountered types in Thailand, a Southeast Asian nation [18]. Similarly, ST23, ST17, ST10, and ST1 constitute the dominant clones in East African countries [19]. In the present study, MLST analysis identified ST19 as the most common sequence type (22.5%), which is consistent with its global prominence in both maternal and adult invasive GBS collections [20,21]. However, the most distinctive feature of the present population was the exclusive clustering of all ST529 isolates within CC327 and their uniform association with serotype V. This ST529–CC327–V combination represented the single most common serotype–ST association in our cohort. A recent WGS study of clindamycin-resistant GBS from Qingdao, Shandong Province, similarly identified V–ST529–CC327 as the predominant lineage, suggesting that this clone may be enriched in coastal areas of Shandong; however, further multicenter studies across eastern coastal China are needed to confirm regional distribution patterns [22]. The phylogenetic inclusion of 70 reference strains revealed that the 40 clinical isolates were distributed across both major GBS lineages, indicating substantial genetic diversity rather than clonal expansion of a single epidemic strain.

While these 40 isolates exhibited substantial phylogenetic diversity, their predicted antimicrobial resistance gene profiles revealed additional clinically relevant patterns. However, as phenotypic antimicrobial susceptibility testing was not performed, all resistance data below reflect genomic predictions rather than confirmed phenotypic resistance. Clinically, GBS remains universally susceptible to penicillin, which continues to serve as the first-line agent for both treatment and prophylaxis [23]. Consistent with established clinical patterns, no predicted penicillin-associated resistance genes were identified in our isolates; however, this does not confirm phenotypic susceptibility, as β-lactam resistance may also arise from penicillin-binding protein mutations or other mechanisms beyond resistance gene detection. Individuals with penicillin allergy must rely on alternative agents such as lincosamides, and the phenotypic resistance burden reported in recent surveillance for these substitutes has become increasingly concerning. Recent studies have demonstrated that resistance to macrolides, lincosamides, and tetracyclines among GBS isolates is rising markedly. European studies report erythromycin and clindamycin resistance at 15–40% (~30%) in pregnant women [24,25], while North America shows higher rates (50.7% and 38.4–43.2%, respectively) [9,26]. China faces a difficult situation, with erythromycin resistance reaching 63% among pregnant women—nearly three times the global average [24]. Broader surveillance of Chinese GBS isolates, predominantly from infant disease cases, further indicates clindamycin and tetracycline resistance exceeding 73% and 98%, respectively [27]. Genomic screening for predicted resistance determinants revealed a high prevalence of macrolide and tetracycline resistance genes. In the present study, the *ermB* gene, which encodes a ribosomal methylase associated with ribosomal methylation-mediated macrolide–lincosamide–streptogramin B resistance, was detected in 60.0% of isolates. It should be emphasized that the presence of resistance genes indicates genomic potential rather than confirmed phenotypic resistance, as macrolide resistance may also result from mutations in 23S rRNA not assessed here. This carriage rate is intermediate between 70.5% in non-pregnant adults from Shandong [28] and 37.5% in pregnant women from Guangzhou [20], but higher than 54.2% in a Bulgarian nationwide collection [29]. Predicted tetracycline resistance genes *tetM* and *tetO* were present in 47.5% and 42.5% of isolates, respectively, comparable to the 46.0% *tetM* carriage in Shandong and *61.1*% in Guangzhou [20,28]. Hierarchical clustering revealed descriptive patterns of predicted resistance gene carriage that varied among STs: ST19 carried *tetM*, *lnuB*, and *lsaE* at high rates (100%, 88.9%, 88.9%) but rarely *ermB/tetO* (11.1%), whereas ST529 showed the inverse pattern (100% *ermB/tetO*, 14.3% *tetM*). Because of the small subgroup sizes, these differences were not formally tested. This dichotomy is consistent with distinct evolutionary pathways hypothesized in previous studies: ST19 potentially acquiring resistance via conjugative transposons of the Tn916 family carrying *tetM* [30,31], and ST529 via integrative conjugative elements (ICEs) harboring *ermB* and *tetO* [22,32]. However, as no mobile genetic element analysis was performed in this study, these proposed mechanisms remain speculative at this stage and require further experimental validation. CC17 exhibited co-carriage of *ermB*, *tetO*, and the aminoglycoside resistance gene cluster *aph(3′)-IIIa/sat4/aad(6)* at 100.0% (3/3), a comprehensive predicted multidrug resistance gene combination characteristic of the hypervirulent CC17 lineage frequently associated with invasive disease [33]. The high co-carriage of *lnuB* and *lsaE* in ST19 (88.9% each) suggests substantial genetic potential for clindamycin resistance, which may limit therapeutic options for penicillin-allergic patients should phenotypic resistance be confirmed [34].

The high overall carriage of predicted virulence-associated genes in this cohort is consistent with the diverse genetic repertoire reported in GBS. The universal presence of *fbsA* and *fbsB* parallels their reported association with fibrinogen-binding [35]. The high carriage of *scpB* and *lmb* (90.0%, 36/40 each) reflects their common genomic occurrence in GBS; in previous work, *lmb* has been associated with laminin-binding and endothelial invasion [36], and *scpB* with C5a peptidase activity that may dampen neutrophil recruitment [37]. Notably, *cylE* and *hylB* were universally present; these genes are predicted to encode β-hemolysin/cytolysin and hyaluronidase, respectively, which have been implicated in membrane disruption and extracellular matrix degradation [38,39]. The *bac* gene was present only in ST10-CC12-Ib isolates (2/2), a descriptive observation given the small sample size. This is consistent with previous reports that *bac* is predominantly associated with serotype Ib; Kong et al. [40] detected Bac in 97% of serotype Ib strains but rarely in other serotypes. The *essC* gene was present in all ST1-CC1-II isolates (4/4). Previous studies have identified *essC* as the ATPase driver of the type VII secretion system (T7SS) in GBS [41]. No hits were obtained for the generic markers *srtC1/C2/C3/C4*, *pilA/B/C*, and *essC* in ST17-CC17 (*n* = 3) and ST1010-CC452 (*n* = 2) isolates. It should be emphasized that all virulence gene data reported herein represent genomic predictions based on in silico annotation against the Virulence Factor Database; locus integrity checks, mutation screening, phenotypic expression, and functional assays were not performed in this study. Consequently, the presence of a given gene does not imply its functional contribution in these isolates. Moreover, the analysis of ST1 and ST17 lineages relied on generic gene screening and did not constitute dedicated PI-1/PI-2a/PI-2b locus typing or full T7SS operon validation; therefore, the presence of *essC* alone cannot be interpreted as evidence for a functional T7SS, and the absence of generic *pilA/B/C* markers should not be considered definitive evidence for the lack of pili in these lineages, particularly given prior reports of high PI-2b carriage in CC17 isolates from a similar region [33].

Several limitations should be acknowledged. This retrospective study used archived genomic DNA without preserved original strains, precluding phenotypic validation of antimicrobial susceptibility or virulence expression. The modest sample size (*n* = 40) and single-province origin limit the precision of subgroup comparisons and preclude formal statistical inference, as well as limiting broader geographic generalizability. The cross-sectional design also precludes assessment of temporal trends. Despite these constraints, the genomic data provide a meaningful snapshot of regional GBS population structure.

## 5. Conclusions

This study describes a genetically diverse GBS population in Zhejiang Province characterized by the prominence of serotype V, the predominance of CC19 followed by CC327, the distinctive ST529–CC327–V clone, and high carriage of predicted macrolide/tetracycline resistance genes and conserved virulence determinants. These findings underscore the importance of ongoing, region-specific genomic surveillance to inform empirical antibiotic stewardship, track the emergence of high-risk clones, and help inform vaccine candidate selection in adult and elderly populations.

## Figures and Tables

**Table 1:** Demographic and clinical characteristics of 40 GBS isolates.

Characteristic	No. (%)
Sex	
Female	28 (70.0)
Male	12 (30.0)
Age group (years)	
0–17	2 (5.0)
18–39	13 (32.5)
40–59	12 (30.0)
≥60	13 (32.5)
Specimen type	
Urine	17 (42.5)
Secretion	14 (35.0)
Blood	5 (12.5)
Tissue fluid	1 (2.5)
Unknown	3 (7.5)

**Table 2:** Serotype distribution of 40 GBS isolates by clinical source.

Serotype	Number of Isolates from Different Clinical Sources (*n* = 40)					Total	Percent (%)
	Urine	Secretion	Blood	Tissue fluid	Unknown		
Ia	1	4	0	1	1	7	17.50
Ib	1	0	1	0	1	3	7.50
II	3	1	0	0	0	4	10.00
III	5	4	1	0	0	10	25.00
IV	0	2	0	0	0	2	5.00
V	7	2	3	0	1	13	32.50
VI	0	1	0	0	0	1	2.50

**Table 3:** Multilocus sequence typing and clonal complex distribution of 40 GBS isolates.

ST	CC	Number of Isolates (*n* = 40)	Percent (%)
1	1	5	12.50
10	12	2	5.00
12	12	1	2.50
17	17	3	7.50
19	19	9	22.50
27	19	1	2.50
23	23	2	5.00
88	23	1	2.50
26	26	1	2.50
485	327	3	7.50
529	327	7	17.50
1010	452	2	5.00
832	/	1	2.50
2662	/	1	2.50
2663	19	1	2.50

Note: /, no available clonal complex data.

## Data Availability

Data are deposited in the National Center for Biotechnology Information (NCBI) BioProject database under accession number PRJNA1517324 (https://www.ncbi.nlm.nih.gov/bioproject/1517324, accessed on 24 August 2026).

## Supplementary Materials

The following supporting information can be downloaded at: https://www.mdpi.com/article/10.3390/microorganisms14102284/s1, Table S1: Overview of genome assembly quality statistics. Table S2: Metadata for the 70 publicly available GBS genomes analyzed in this study. Table S3: Binary presence matrix of antimicrobial resistance and virulence genes of 40 GBS isolates. Table S4: The clinical metadata of 40 GBS isolates.
